# Gender specific influence of serotonin on core symptoms and neurodevelopment of autism spectrum disorders: A multicenter study in China

**DOI:** 10.1186/s13034-025-00892-7

**Published:** 2025-03-29

**Authors:** Qiu-hong Mou, Qian Zhang, Li Chen, Ying Dai, Hua Wei, Fei-Yong Jia, Yan Hao, Ling Li, Jie Zhang, Li-Jie Wu, Xiao-Yan Ke, Ming-Ji Yi, Qi Hong, Jin-Jin Chen, Shuan-Feng Fang, Yi-Chao Wang, Qi Wang, Jie Chen, Ting-Yu Li, Ting Yang

**Affiliations:** 1 Growth, Development and Mental Health Center of Children and Adolescents, Chongqing Key Laboratory of Child Neurodevelopment and Cognitive Disorders, Ministry of Education Key Laboratory of Child Development and Disorders, National Clinical Research Center for Child Health and Disorders, Children’S Hospital of Chongqing Medical University, Chongqing, China; 2 Department of developmental and behavioral pediatrics, The First Hospital of Jilin University, Changchun, China; 3 Department of Pediatrics, Tongji Hospital, Tongji Medical College, Huazhong University of Science and Technology, Wuhan, China; 4 Department of Children Rehabilitation, Hainan Women and Children’S Medical Center, Haikou, China; 5 Children Health Care Center, Xi’an Children’S Hospital, Xi’an, China; 6 Department of Children’S and Adolescent Health, Public Health College of Harbin Medical University, Harbin, China; 7 Child Mental Health Research Center of Nanjing Brain Hospital, Nanjing, China; 8 Department of Child Health Care, The Affiliated Hospital of Qingdao University, Qingdao, China; 9 Maternal and Child Health Hospital of Baoan, Shenzhen, China; 10 Department of Child Healthcare, Shanghai Children’S Hospital, Shanghai Jiao Tong University, Shanghai, China; 11 Children’S Hospital Affiliated to Zhengzhou University, Zhengzhou, China; 12 NHC Key Laboratory of Birth Defect for Research and Prevention, Hunan Provincial Maternal and Child Health Care Hospital, Changsha, China; 13 Deyang Maternity & Child Healthcare Hospital, Deyang, Sichuan China

**Keywords:** Autism spectrum disorder, 5-HT, Neurological development, Clinical symptoms, Sex differences

## Abstract

**Background:**

High serotonin (5-hydroxytryptamine [5-HT]) blood levels are the most reliable and frequently replicated biomarker for autism spectrum disorders (ASDs). However, their differential influence on core ASD symptoms in males and females remains unclear. This study aimed to investigate the changes in 5-HT levels in children with ASD to assess and compare its influence on the core symptoms and neurodevelopment of boys and girls.

**Methods:**

Herein, 1,457 ASD children and 1,305 typically developing (TD) controls (age = 2–7 years) were enrolled from 13 cities across China. Social Responsiveness Scale (SRS) and Childhood Autism Rating Scale (CARS) were used to evaluate the ASD symptoms in children, and the revised Children Neuropsychological and Behavior Scale-Revision 2016 (CNBS-R2016) was used to evaluate their neurodevelopment. The 5-HT serum levels were measured by high-performance liquid chromatography-tandem mass spectrometry.

**Results:**

In boys with ASD, increased serum 5-HT levels correlated with high scores on SRS and CARS and with communication warning behavior of CNBS-R2016. Conversely, concomitant decline was observed in the scores on the general, language, gross motor, adaptive behavior, and personal-social quotients. Notably, no differences were found in girls with ASD.

**Conclusions:**

Children with ASD, especially boys, presented higher serum 5-HT levels compared with TD children. Additionally, increased 5-HT content is considerably positively associated with core ASD symptoms and negatively associated with neurodevelopment in boys with ASD. Overall, this study highlights the gender bias in patients with ASD regarding 5-HT serum levels, underscoring its influence on ASD prevalence in a sex-specific manner.

**Trial registration:**

This study has been approved by the Ethics Committee of the Children’s Hospital of Chongqing Medical University (approval number: (2018) IRB (STUDY) NO.121). Additionally, this study is registered with the China Clinical Trial Registry (Registration Number: ChiCTR2000031194).

**Supplementary Information:**

The online version contains supplementary material available at 10.1186/s13034-025-00892-7.

## Introduction

Autism spectrum disorder (ASD) is a complex and heterogeneous neurodevelopmental disorder, with a prevalence of approximately 2.7% (1/36) in children aged 8 years in the United States, and a global prevalence of 1–2% [20, 21, 35]. In 2019, a survey was carried out with 125,806 children aged 6–12 years across eight major cities in China, and a 0.7% prevalence of ASD was reported, with prevalence in males being four–five times that of females [36]. Such notable differences in ASD prevalence between sexes highlight the importance of understanding the role of sex-related biological factors in ASD [17]. Many studies have been conducted on early diagnosis of ASD in clinical settings by comparing differential biomarkers between male and female children with ASD.

Serotonin (5-hydroxytryptamine [5-HT]), biosynthesized from tryptamine, is an important hormone and neurotransmitter that is implicated in various neurobiological functions in the central nervous system. More than 25% of individuals with ASD have been reported to exhibit hyperserotonemia, and high blood 5-HT levels have been identified as the first biomarker for autism [11]. Many studies have been conducted on comparing blood 5-HT levels between healthy individuals and those with ASD [30], and correlating 5-HT levels with core symptoms, neurocognition, emotional behavior, or related clinical outcomes of ASD. However, inconsistencies have been reported regarding peripheral blood 5-HT levels and clinical outcomes of ASD, citing limitations in sample size, biomaterials, and methods [8]. Studies have indicated a higher prevalence of ASD in males compared to females [10]. Despite hyperserotonemia has been commonly observed in children with ASD, sex-specific variations in 5-HT levels and their clinical implications remain underexplored. A seminal investigation involving 182 prepubertal ASD children revealed significant elevated rates of hyperserotonemia in males compared to females [31]. Conversely, other research indicated that while platelet 5-HT levels were significantly higher in ASD children compared to TD children, there was no notable difference between male and female children with ASD [4]. These inconsistent findings highlight the necessity for comprehensive multicenter studies aimed at systematically characterizing sex-based differences in 5-HT regulation and assessing their potential role in modulating core symptoms of ASD.

This multicenter, large-sample, cross-sectional study—based on the China Multi-Center Preschool Autism Project (CMPAP)—represented a significant advancement over previous research. Notably, it included the largest sample to date for exploring gender differences in 5-HT levels between children with ASD and TD controls. With 1,457 children with ASD and 1,305 TD controls, the study provided unparalleled statistical power and generalizability. Unlike prior studies that were limited by smaller sample sizes or single-center designs, this investigation aimed to: (1) examine the differences in peripheral 5-HT levels between children with ASD and TD children; (2) identify correlations between 5-HT dysregulation and core clinical symptoms; and (3) explore sex-mediated differences in 5-HT and their association with the core clinical manifestations of ASD.

## Methods

### Design, participants and procedure

**Fig. 1:**
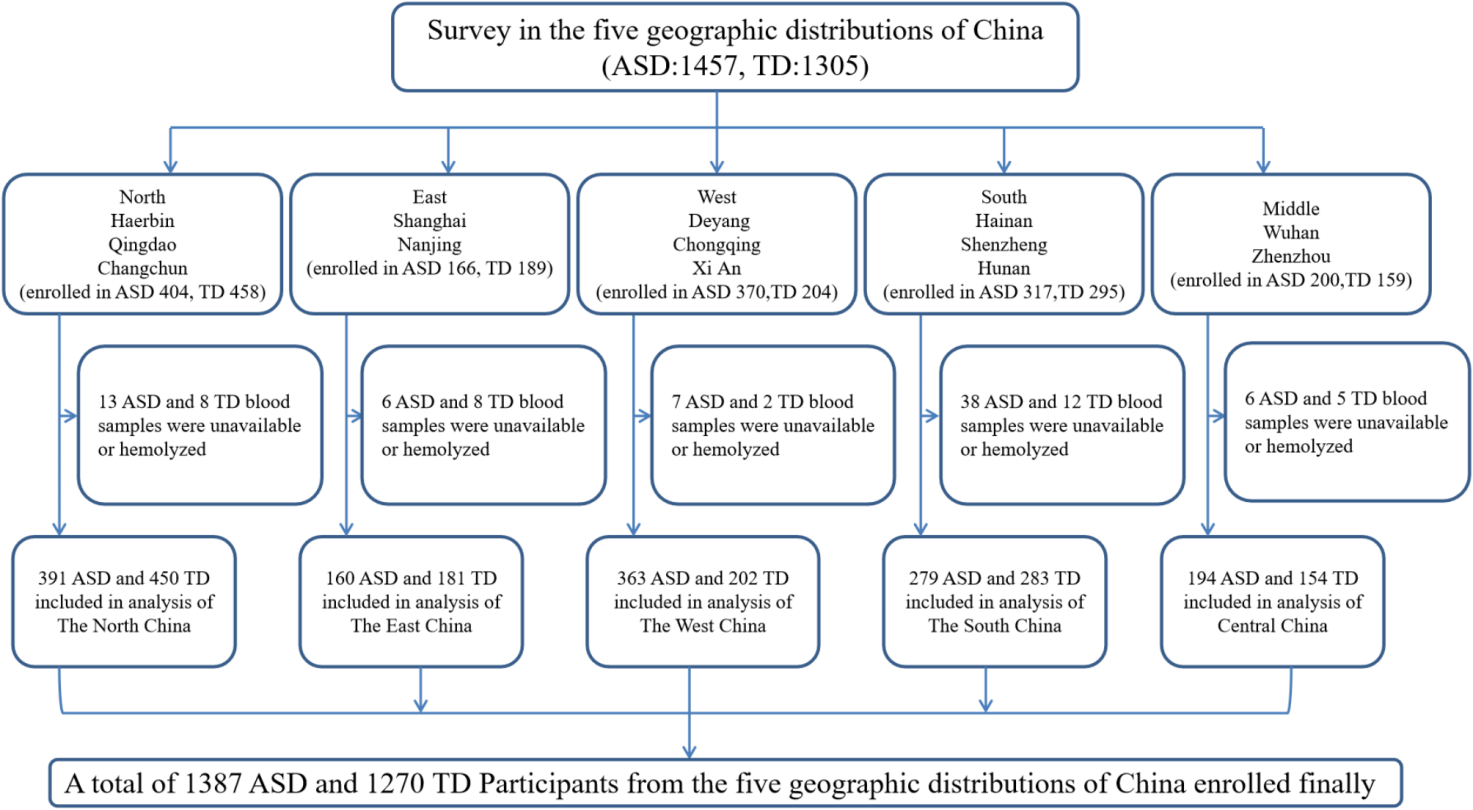
Flow diagram illustrating the survey profile. *ASD* autism spectrum disorder, *TD* typically developing

The nationwide multi-center, cross-sectional survey was carried out as a sub-project of the CMPAP sponsored by the Subspecialty Group of Developmental and Behavioral Pediatrics of the Society of Pediatrics, Chinese Medical Association [34]. The subjects for the survey were recruited from May 2018 to December 2019 from 13 cities across the five major regions of China: northern region (Harbin, Qingdao, and Changchun), eastern region (Shanghai and Nanjing), western region (Chongqing, Deyang, and Xi’an), southern region (Shenzhen, Haikou, and Changsha), and central region (Wuhan and Zhengzhou). In total, 2,762 children (age = 2–7 years) were recruited in the survey, including 1,457 children with ASD and 1,305 typically developing (TD) children. Excluding the cases where blood samples could not be collected or exhibited hemolysis, 1,387 children with ASD were compared to 1270 TD children in the final analysis. Figure 1 presents the flow chart of the screening process. All ASD diagnoses were performed by developmental pediatricians at local hospitals after various structured interviews based on the criteria for ASD defined in the Diagnostic and Statistical Manual of Mental Disorders, 5th Edition (DSM-5). Each diagnosis was assessed using a Children’s Autism Rating Scale (CARS). Exclusion criteria for children with ASD were as follows: (1) those with an etiologically known genetic or neurological disease (such as fragile X, Rett syndrome); (2) those with severe sensory or motor impairment; (3) those with major physical disease; (4) those with chronic epilepsy; and (5) those with history of severe head injury. TD children were recruited from local kindergartens or online, and they presented normal growth and development histories, with no ASD diagnosis history among first- or second-degree relatives. Individuals with a history of severe head injury were excluded.

All children enrolled voluntarily in this study, and informed consent was signed by their guardians. This study has been approved by the Ethics Committee of the Children’s Hospital of Chongqing Medical University (approval number: (2018) IRB (STUDY) NO.121). Additionally, this study is registered with the China Clinical Trial Registry (Registration Number: ChiCTR2000031194).

### Questionnaire and measures

Questionnaire information was collected by uniformly trained investigators via face-to-face interviews with the guardians of children with ASD and TD children, such as basic demographic data (including name, gender, place of residence, and ethnicity), disease history, and medication history.

Physical measurements of children with ASD and TD children were recorded by trained professionals using electronic weights and height gauges. The weight height measurement values were accurate to 0.05 kg and 0.1 cm, respectively. The average value of three measurements was selected. The raw values were normalized according to age and gender using the World Health Organization Anthro and AnthroPlus software (World Health Organization, 2009; Anthro for Personal Computers, Version 3.01: Software for Assessing Growth and Development of the World’s Children).

### Clinical symptom assessment

Specialist physicians diagnosed ASD in children based on the DSM-5 ASD criteria and clinical symptoms. Herein, one parental assessment scales, namely the Social Responsiveness Scale (SRS) and CARS, a specialist assessment scale operated by developmental pediatricians, were used to evaluate the clinical symptoms. Additionally, the neurodevelopment in children was assessed using the Neuropsychological and Behavior Scale-Revision 2016 (CNBS-R2016). The assessors underwent standardized operation training on symptom assessment scales before implementing the project to ensure the accuracy of symptom assessment.

The SRS mainly evaluates the impairment of social function in children and is widely used for ASD screening. It comprises a total of 65 items and includes the following five sub-scales: social awareness, social motivation, social communication, social cognition, and autistic mannerisms. Each item was rated on a scale of 1–4 based on the performance of the child over the past 6 months. A score of < 65 points represents normal development in children [3,5].

The CARS is widely used as a professional behavioral observation tool for determining the severity of autism symptoms. It contains a total of 15 items, with each item being scored on a scale of 1–4 based on severity (total score = 60) [27, 29]. A total score of < 30 points means no autism; 30–36 points denote mild to moderate autism; and a total score of ≥ 37 points and at least five items with > 3 points denote severe autism.

The CNBS-R2016 was used to assess the neurodevelopment level of children with ASD. It includes the following five components: gross motor, fine motor, adaptive behavior, language, and personal social skills; additionally, a separate subscale, namely Communication Warning Behavior, was added to assess autism symptoms. A general quotient (GQ) or subscale development quotient of < 70 (< 2 standard deviations [22]) indicated developmental delay (DD). In the Communication Warning Behavior subscale, a score of 12–30 indicated a risk of communication and interaction barriers, and that of > 30 indicated a high suspicion of ASD. Reportedly, the CNBS-R2016 exhibits good consistency with the Griffiths Mental Development Scales. Herein, the effective samples for SRS, CARS, and CNBS-R2016 were 1,177, 1,178, and 992, respectively [18].

### Blood sample processing and serum 5-HT detection

Herein, 5 mL of venous blood was collected in dry red-tipped blood tubes without additives. Within 1 h of collection, the samples were centrifuged (3,000 rpm, 5 min), and the separated serum was transferred to a microcentrifuge tube and stored at −80 °C. All samples were sent for 5-HT level detection within one week of collection. To maintain sample integrity, repeated freeze-thaw cycles were avoided, and all samples were kept protected from direct light exposure throughout the process.

Next, 100 µL of serum samples or quality controls were prepared by adding 10 µL of internal standards and 500 µL of methyl alcohol, which were then vortex-mixed for 3 min and centrifuged at 14,000 rpm for 10 min. Following this, 50 µL of supernatant was isolated and added with 200 µL of ultrapure water and subjected to vortex mixing for 30 s. Finally, the samples were detected by high-performance liquid chromatography-tandem mass spectrometry (HPLC-MS/MS; detection instrument model: LC1260/MSK6460C, Agilent Company, USA).

### Statistical analyses

The Statistical Package for Social Sciences 22.0 software (IBM Corporation, Armonk, NY, USA) was used for statistical analyses. Continuous variables were analyzed using mean, SD, median, and interquartile range (IQR). Categorical variables were analyzed using frequency and percentages per category. Kolmogorov-Smirnov test was used to test whether the continuous variables conform to a normal distribution, and Mann–Whitney test was used to compare the differences between two groups for continuous variables that did not conform to the normal distribution. Categorical variables were compared using Chi-square tests. Generalized linear models (GLM) with gamma regression was performed for 5-HT levels in ASD and TD groups; gender-specific 5-HT levels in TD children and those with ASD; 5-HT levels in children with ASD regarding different gender and severity; and 5-HT levels among ASD boys with different neurodevelopmental levels, with age as a covariate. Additionally, multivariate linear regression models were separately used for boys and girls, with age being a covariate, to compare the correlations of 5-HT levels on SRS, CARS, and CNBS-R2016 scores in the ASD group. Associations were determined using the odds ratio (OR) and 95% confidence interval (CI). A two-sided *P*-value of < 0.05 was considered statistically significant.

## Results

### Demographic and physical characteristics of TD and ASD groups

The demographic and physical development features of enrolled subjects are presented in Table 1. The results revealed significant differences in both age and gender distribution between the ASD and TD groups (*P* < 0.001). To account for these differences, subsequent analyses were stratified by gender and adjusted for age. Notably, the Z-scores of weights and body mass index between the two groups in either boys or girls were not significantly different, whereas the height of children with ASD was significantly higher than that for TD children (Table 1).

**Table 1:** Demographic characteristics of the participants in TD and ASD groups

Variable	TD (*n* = 1270)	ASD (*n* = 1387)	Z/chi-square	*P*
Age (years), Median (IQR)	4.42 (3.38–5.38)	3.96 (3.13–4.90)	− 6.290	<0.001
**Gender, n(%)**				
Boys	832 (65.51)	1137 (81.98)	93.640	<0.001
Girls	438 (34.49)	250 (18.02)		
**Boys**	*n* = 806	*n* = 952		
Z_HA_, Median (IQR)	0.01 (− 0.64, 0.81)	0.24 (− 0.52, 0.93)	− 2.248	0.025
Z_WA_, Median (IQR)	0.27 (− 0.38, 0.88)	0.36 (− 0.35, 1.05)	− 1.251	0.211
Z_BMIA_, Median (IQR)	0.30 (− 0.41, 1.09)	0.26 (− 0.44, 1.09)	− 0.568	0.570
**Girls**	*n* = 420	*n* = 204		
Z_HA_, Median (IQR)	0.06 (− 0.66, 0.65)	0.26 (− 0.52, 0.98)	− 2.437	0.015
Z_WA_, Median (IQR)	0.17 (− 0.48, 0.73)	0.22 (− 0.45, 0.85)	− 0.883	0.377
Z_BMIA_, Median (IQR)	0.15 (− 0.50, 0.82)	0.03 (− 0.61, 0.76)	− 1.049	0.294

Data was shown as Median (IQR) or number (percentage). Chi-square test and Mann-Whitney U test were used in the analysis

*ASD* autism spectrum disorder, *TD* typically developing, *IQR* interquartile range, *Z*_*HA*_ Z-score of Height-for-Age, *Z*_*WA*_ Z-score of Weight-for-Age, *Z*_*BMIA*_ Z-score of BMI-for-Age

## Higher serum 5-HT levels in the ASD group, especially in boys

The GLM, with age as a covariate, was performed to analyze serum 5-HT levels between the ASD and TD groups, as well as between different genders within these groups. Compared with the TD group, the ASD group presented significantly higher serum 5-HT levels (*P* = 0.001); a similar difference was observed for 5-HT serum levels in boys between ASD and TD groups (*P* = 0.006). However, serum 5-HT levels in girls did not differ significantly between the two groups (*P* = 0.452). Furthermore, serum 5-HT levels in boys of the ASD group were significantly higher than those in the girls (*P* = 0.004) (Table 2). The findings suggested that elevated serum 5-HT levels may be associated with ASD, particularly in boys, highlighting potential gender-specific differences in 5-HT regulation.

**Table 2:** Comparison of the levels of serum 5-HT between children with ASD and TD

Group total	5-HT, Median (IQR)	Wald chi-square	*P*-value
TD (*n* = 1270)	132.00 (99.75–170.00)	12.108	0.001
ASD (*n* = 1387)	136.00 (97.00–180.00)		
**Boys**			
TD (*n* = 832)	134.00 (103.00–173.75)	7.494	0.006
ASD (*n* = 1137)	137.00 (99.00–182.00)		
**Girls**			
TD (*n* = 438)	126.00 (91.00–166.00)	0.566	0.452
ASD (*n* = 250)	128.00 (91.75–167.50)		
**Gender**			
Boys with ASD (*n* = 1137)	137.00 (99.00–182.00)	8.076	0.004
Girls with ASD (*n* = 250)	128.00 (91.75–167.50)		

Generalized linear models (GLM) with gamma regression were employed to compare the serum 5-HT levels between the two groups, with age as covariate

*TD* typically developing, *ASD* autism spectrum disorder, *IQR* interquartile range

### Higher serum 5-HT levels in children with severe ASD, especially in boys

The GLM, with age being a covariate, for boys and girls with mild-moderate to severe ASD revealed that children with severe ASD exhibited higher serum 5-HT levels than children with mild-moderate ASD (*P* = 0.003). This trend was particularly evident in boys, with boys exhibiting severe ASD demonstrating significantly higher 5-HT levels compared to those with mild-to-moderate ASD. (*P* = 0.002), whereas no differences were found between the girls of the two groups (*P* = 0.847) (Table 3). These findings suggested that the severity of ASD may be associated with elevated serum 5-HT levels, particularly in male subjects, indicating potential gender-specific biological mechanisms underlying ASD progression.

**Table 3:** Comparison of the levels of serum 5-HT in children with ASD of different symptom severity

Group	5-HT, Median (IQR)	Wald chi-square	*P*-value
**Total**			
Mild-moderate ASD (*n* = 829)	135.00 (97.00–179.00)	8.831	0.003
Severe ASD (*n* = 349)	142.00 (103.00–199.5)		
**Boys**			
Mild-moderate ASD (*n* = 685)	136.00 (97.00–180.50)	9.674	0.002
Severe ASD (*n* = 290)	145.00 (106.25–205.25)		
**Girls**			
Mild-moderate ASD (*n* = 144)	127.00 (98.00–174.25)	0.037	0.847
Severe ASD (*n* = 59)	130.00 (92.00–161.00)		

Generalized linear models (GLM) with gamma regression were employed to compare the serum 5-HT levels between the two groups, with age as covariate

*ASD* autism spectrum disorder, *IQR* interquartile range

### Association between 5-HT levels and gender-specific clinical assessment scale scores of children with ASD

With age as a covariate, a multivariate linear regression model was used to analyze the correlations of serum 5-HT levels on scores of SRS, CARS, and Communication Warning Behavior subset of CNBS-R2016. Notably, Children with ASD who exhibited higher serum 5-HT levels demonstrated higher scores on social awareness (β = 1.822, 95% CI: 0.691–2.953; *P* = 0.002), social cognition (β = 1.095, 95% CI: 0.274–1.916; *P* = 0.009), autistic mannerisms (β = 0.668, 95% CI: 0.036–1.301; *P* = 0.038), and total of SRS (β = 0.211, 95% CI: 0.047–0.375; *P* = 0.012); higher total scores of CARS (β = 1.110, 95% CI: 0.561–1.659; *P <* 0.001); and higher scores of Communication Warning Behavior of CNBS-R2016 (β = 0.311, 95% CI: 0.112–0.510; *P* = 0.002). Additionally, serum 5-HT levels positively correlated with the social awareness, social cognition, social communication, social motivation and total scores of SRS, CARS, and the Communication Warning Behavior of CNBS-R2016 in boys. These correlations were significant in boys but not in girls, with detailed gender-specific differences presented in Table 4.

**Table 4:** Correlations of serum 5-HT levels on the scores of SRS, CARS and communication warning behavior of CNBS-R2016 in the ASD children at different sex groups

Behavior subscales	Total		Boys		Girls	
	β (95% CI)	*P*	β (95% CI)	*P*	β (95% CI)	*P*
**SRS scale scores**	*n* = 1177		*n* = 966		*n* = 211	
Social awareness	1.822 (0.691 to 2.953)	0.002	2.080 (0.814 to 3.345)	0.001	0.888 (− 1.579 to 3.355)	0.479
Social cognition	1.095 (0.274 to 1.916)	0.009	1.188 (0.276 to 2.099)	0.011	0.700 (− 1.159 to 2.559)	0.459
Social communication	0.374 (− 0.046 to 0.795)	0.081	0.511 (0.039 to 0.983)	0.034	− 0.176 (− 1.081 to 0.728)	0.701
Social motivation	0.576 (− 0.169 to 1.321)	0.130	0.889 (0.054 to 1.725)	0.037	− 0.724 (− 2.328 to 0.881)	0.375
Autistic mannerisms	0.668 (0.036 to 1.301)	0.038	0.686 (− 0.020 to 1.392)	0.057	0.484 (− 0.914 to 1.883)	0.496
Total score	0.211 (0.047 to 0.375)	0.012	0.257 (0.074 to 0.440)	0.006	0.013 (− 0.347 to 0.373)	0.944
**CARS scale scores**	*n* = 1178		*n* = 975		*n* = 203	
Total score	1.110 (0.561 to 1.659)	<0.001	0.290 (0.660 to 1.920)	<0.001	0.331 (− 0.737 to 1.399)	0.542
Communication Warning Behavior	*n* = 982		*n* = 801		*n* = 181	
	0.311 (0.112 to 0.510)	0.002	0.389 (0.164 to 0.613)	0.001	− 0.004 (− 0.415 to 0.407)	0.984

Multivariate Linear Regression Models was employed to analyze the association between serum 5-HT level and scores of scales, with age as covariate

*SRS* Social Responsiveness Scale, *CARS* Childhood Autism Rating Scale, *CNBS-R2016* Children Neuropsychological and Behavior Scale-Revision 2016, *ASD* autism spectrum disorder, *β (95%CI)* regression coefficient (95% confidence interval)

### Boys with ASD presenting high 5-HT levels had higher scores for clinical symptoms

The multiple linear regression model was applied after dividing the 5-HT levels in boys with ASD into three subgroups. After adjusting for age covariates, the relationship between 5-HT levels and clinical scale scores was examined. With the first quartile as reference data, the third quartile of ASD presented higher SRS scores on social awareness (OR = 1.075, 95% CI: 1.026–1.127; *P* = 0.003), social cognition (OR = 1.037, 95% CI: 1.003–1.073; *P* = 0.034), social motivation (OR = 1.033, 95% CI: 1.002–1.066; *P* = 0.038), autistic mannerisms (OR = 1.027, 95% CI: 1.001–1.054; *P* = 0.045) and SRS total scores (OR = 1.009, 95% CI: 1.002–1.016; *P* = 0.010), CARS total scores (OR = 1.037, 95% CI: 1.014–1.061; *P* = 0.002), and Communication Warning Behavior of CNBS-R2016 scores (OR = 1.010, 95% CI: 1.002–1.018; *P* = 0.014) (Table 5). These data demonstrated that elevated 5-HT levels are associated with increased severity of various clinical symptoms in male subjects with ASD.

**Table 5:** Estimated associations between serum 5-HT levels and the scores of SRS, CARS and communication warning behavior of CNBS-R2016 in the ASD boys

Behavior subscales	Quartiles 1 (≤ 113 ng/ml)	Quartiles 2 (113–165 ng/ml)	*P*	Quartiles 3 (>165 ng/ml)	*P*
	OR (95% CI)	OR (95% CI)		OR (95% CI)	
**SRS scale scores**					
Social awareness	Reference	1.060 (1.011 to 1.112)	0.016	1.075 (1.026 to 1.127)	0.003
Social cognition	Reference	1.016 (0.982 to 1.051)	0.363	1.037 (1.003 to 1.073)	0.034
Social communication	Reference	1.014 (0.997 to 1.032)	0.116	1.017 (1.000 to 1.035)	0.053
Social motivation	Reference	1.028 (0.997 to 1.061)	0.079	1.033 (1.002 to 1.066)	0.038
Autistic mannerisms	Reference	1.015 (0.989 to 1.042)	0.271	1.027 (1.001 to 1.054)	0.045
Total score	Reference	1.006 (1.000 to 1.013)	0.069	1.009 (1.002 to 1.016)	0.010
**CARS scale scores**					
Total score	Reference	1.018 (0.995 to 1.042)	0.135	1.037 (1.014 to 1.061)	0.002
Communication Warning Behavior	Reference	1.000 (0.992 to 1.008)	0.972	1.010 (1.002 to 1.018)	0.014

Multivariate logistics regression was used for were employed to analyze the association between serum 5-HT level and scores of scales, with age as covariate

*SRS* Social Responsiveness Scale, *CARS* Childhood Autism Rating Scale, *CNBS-R2016* Children Neuropsychological and Behavior Scale-Revision 2016, *ASD* autism spectrum disorder, *OR* odds ratio,*CI* confidence interval

### Associations between 5-HT and gender-specific neurodevelopment of children with ASD

The neurodevelopmental variables were markedly associated with 5-HT levels in children with ASD, particularly boys. The 5-HT levels were negatively associated with the GQ (β = −0.297, 95% CI: −0.523 to −0.071, *P* = 0.010), gross motor quotient (β = −0.301, 95% CI: −0.503 to −0.098, *P* = 0.004), adaptive behavior (β = −0.209, 95% CI: −0.411 to −0.007, *P* = 0.043), and personal-social quotient (β = −0.255, 95% CI: −0.458 to −0.053, *P* = 0.013) in children with ASD. Additionally, the serum 5-HT levels and gender-specific development quotients in the ASD group showed a negative correlation for the GQ, gross motor, adaptive behavior, language, and personal-social quotient in boys with ASD. Notably, no difference was observed for girls with ASD. The differences in each neurodevelopmental quotient are presented in Table 6. These results suggested that elevated 5-HT levels may be associated with poorer neurodevelopmental outcomes, particularly in boys with ASD. The gender-specific differences highlighted the potential influence of biological sex on the relationship between 5-HT and neurodevelopment in ASD.

**Table 6:** Effect of serum 5-HT levels on the scores of CNBS-R2016 in the ASD children at different sex groups

CNBS-R2016	Total(*n* = 992)		Boys(*n* = 811)		Girls(*n* = 181)	
	β (95% CI)	*P*	β (95% CI)	*P*	β (95% CI)	*P*
Gross motor	− 0.301 (− 0.503 to − 0.098)	0.004	− 0.309 (− 0.540 to − 0.077)	0.009	− 0.271 (− 0.667 to 0.125)	0.178
Fine motor	− 0.210 (− 0.425 to 0.005)	0.055	− 0.243 (− 0.487 to 0.001)	0.051	− 0.111 (− 0.545 to 0.324)	0.615
Adaptive behavior	− 0.209 (− 0.411 to − 0.007)	0.043	− 0.255 (− 0.487 to − 0.023)	0.031	− 0.075 (− 0.468 to 0.319)	0.708
Language	− 0.149 (− 0.313 to 0.015)	0.074	− 0.213 (− 0.399 to − 0.027)	0.025	0.094 (− 0.241 to 0.429)	0.579
Personal-social	− 0.255 (− 0.458 to − 0.053)	0.013	− 0.285 (− 0.516 to − 0.054)	0.016	− 0.116 (− 0.516 to 0.283)	0.565
Genernal quotient	− 0.297 (− 0.523 to − 0.071)	0.010	− 0.352 (− 0.610 to − 0.094)	0.008	− 0.109 (− 0.554 to 0.336)	0.629

Univariate Logistics Regression was employed to analyze the association between serum 5-HT level and scores of CNBS-R2016 scale, with age as covariate

*CNBS-R2016* Children Neuropsychological and Behavior Scale-Revision 2016, *ASD* autism spectrum disorder, *β (95%CI)* regression coefficient (95% confidence interval)

### Boys with ASD exhibiting moderate-severe DD had higher 5-HT levels

The boys with ASD were divided into three subgroups based on the GQ: ASD without DD, GQ ≥ 70; ASD with mild DD, 55 ≤ GQ < 70; and ASD with moderate-severe DD, GQ ≤ 54. There was a significant difference of serum 5-HT levels among these three subgroups (*P* = 0.043) (Table 7). In addition, pairwise comparisons revealed that 5-HT levels in the ASD with moderate-severe DD subgroup were significantly higher than that in the ASD without DD subgroup (*P* = 0.015), and there were no differences between ASD with mild DD and other two groups. These results suggested that elevated 5-HT levels may be associated with more severe developmental delays in boys with ASD, highlighting a potential link between 5-HT dysregulation and the severity of neurodevelopmental impairments.

**Table 7:** The serum 5-HT levels among ASD boys with different neurodevelopmental levels

Scale scores	5-HT Median (IQR)	Wald chi-square	*P*-value
ASD without DD (*n* = 259)	133.00 (95.00–181.00)	6.304	0.043
ASD with mild DD (*n* = 264)	137.00 (98.00–180.00)		
ASD with moderate-severe DD (*n* = 288)	149.00 (103.25–198.75)*		

Generalized linear models (GLM) with gamma regression were employed to compare the serum 5-HT levels between the three groups, with age as covariate. “*” represents a significant difference between the ASD group without DD and the ASD group with moderate-severe DD, *P* = 0.015

*DD* development disorder, *ASD* autism spectrum disorder, *IQR* interquartile range

## Discussion

Over the past few decades, ASD, a rare disorder, has been included among the most commonly studied and publicized neurodevelopmental disorders [35]. Owing to its high heterogeneity, ASD screening and diagnosis are primarily based on behavioral observations by professionals, which contribute to low early screening and diagnosis rates. Consequently, research on ASD biomarkers and their sex-associated differences has garnered notable attention [8]. Hyperserotonemia has been identified as a biological endophenotype of ASD, with a partial hereditary association [2]. However, despite extensive studies, underlying reasons associated with high 5-HT levels in patients with ASD and their association with clinical symptoms and sex-associated differences remain unelucidated. To our knowledge, this is the first nationwide multicenter cross-sectional study investigating the sex-specific association between 5-HT levels and clinical symptoms in children with ASD. Notably, our study revealed significant gender-associated differences in serum 5-HT levels in children with ASD, with boys exhibiting a greater influence on core symptoms and neuropsychological development. These findings provide new insights into the 5-HT mediated pathophysiological mechanisms that contribute to the heterogeneity of ASD. Furthermore, these suggest potential neurobiological bases for the observed sex differences in ASD clinical manifestations.

Increased peripheral blood 5-HT levels or hyperserotonemia is the first identified biomarker of ASD, affecting over 25% of children with ASD [25], which may be attributed to its involvement in various brain systems [28]. Recently, many reviews have associated high 5-HT with ASD [8, 11]. Recent studies have demonstrated elevated 5-HT levels in individuals with ASD [8, 19, 23,24], although systematic analyses of 113 studies reveal inconsistent findings, with 19 studies reporting either reduced 5-HT levels or no significant differences compared to TD controls [8]. Moreover, variations in age distribution among study populations have been recognized as a potential confounding factor when measuring peripheral 5-HT levels [26, 31]. In this study, after implementing rigorous age-adjustment procedures, we have confirmed previous findings showing elevated 5-HT levels in children with ASD. Our results indicated that pediatric ASD cases demonstrate significantly increased blood serotonin levels, which might potentially result from gastrointestinal hypersecretion [14]. The overproduction of 5-HT may be due to genetic factors, especially mutations in the TPH and SERT genes, which disrupt normal 5-HT synthesis and reuptake. Additionally, environmental and physiological factors such as infections, gut microbiota dysbiosis, and immune system dysfunction may also contribute to 5-HT dysregulation in ASD individuals [7, 9].

Owing to reported gender-specific differences in ASD prevalence, it is crucial to understand the role of biological sex-related biomarkers in ASD prevalence [17]. However, studies on sex-related differences in peripheral blood 5-HT levels in children with ASD are limited. Our sex-stratified analysis revealed significantly higher 5-HT levels in boys with ASD compared to TD boys. while no significant difference between the two groups of girls. Consistent with a previous study in India, which found that platelet 5-HT levels in ASD cases were 2.5 times higher than in controls. Additionally, male children with ASD had significantly elevated levels, while no significant difference was observed in females [4]. Furthermore, 5-HT levels were notably higher in boys compared with that in the girls in the ASD group. Previous research involving 182 preadolescent children with ASD found that 42% of the participants exhibited hyperserotonemia, with males were significantly more affected than females. However, that study was limited by insufficient sample size for robust sex comparisons and lack of a TD group [31]. Our current study addresses these limitations by employing an expanded sample size and including TD controls, thereby enabling more robust and comprehensive comparisons. The observed gender disparity in ASD prevalence may result from interactions among genetic factors, sex hormones, and neurodevelopment. Males are particularly susceptible due to sex-linked inheritance patterns: both the MAOA and SERT genes are on the X chromosome. A defective allele on the X chromosome, such as a low-activity MAOA variant, disrupts 5-HT degradation in males, leading to elevated peripheral blood 5-HT levels. Additionally, rare variants of the SERT gene enhance 5-HT uptake specifically in males [25]. The hormonal influence on serotonergic function further contributes to this gender-specific manifestation. Estrogen has been shown to regulate serotonergic pathways, thereby influencing emotional and cognitive processes [12]. These effects, combined with inherent differences in brain organization between genders, result in distinct behavioral phenotypes. Collectively, these factors contribute to the gender-specific association between elevated 5-HT levels and ASD-related behavioral abnormalities.

Current research indicates that investigating the relationship between 5-HT and core symptoms or other clinical outcomes of ASD is challenging. Future studies need to consider the individual effects of 5-HT on core symptoms and neurocognitive and neuropsychological profiles of ASD [8]. Our findings demonstrated a positive correlation between alterations in 5-HT levels and the severity of core ASD symptoms. Specifically, an increase in 5-HT content was associated with an increase in scores across different subscales and total scores of SRS, CARS, and Communication Warning Behavior of CNBS-R2016, along with the concomitant decline in neurocognitive response scores, especially in boys with ASD. The results of this study are consistent with those of most studies [1, 13, 33]; however, some studies have shown that high 5-HT levels in children with ASD are not correlated with the severity of overall phenotypic expression according to the CARS or ADOS scores [4, 22]. Additionally, 5-HT levels in ASD participants have been reported to be inversely correlated with neurodevelopmental response levels, especially in male participants [4], which is consistent with the findings of this study that support the possible involvement of 5-HT in ASD pathogenesis, diagnosis, and severity assessment for prognostic purposes. Social behaviors could be regulated by neurotransmitters, such as 5-HT. A randomized controlled trial revealed that administering buspirone, a partial 5-HT receptor 1 A agonist, reduced repetitive and restricted behavior in ASD [6]. And the serotonergic system has been reported to contribute to regulate social behavior and cognition via serotonergic receptors [16, 32]. The increased 5-HT levels in the peripheral blood and presynaptic areas may serve as a compensatory mechanism to maintain its concentration in circulation or synapses, where 5-HT performs its function. Notably, an association between ASD and the SERT gene has been reported [15]. Consequently, genetic factors affecting serotonergic system genes may facilitate the regulation of 5-HT function and homeostasis, which are modulated differently in males and females with ASD. These findings suggest that 5-HT influences behavioral phenotypes differently in boys with ASD compared to girls, potentially through inherited mechanisms [4].

This study has several limitations that should be acknowledged. First, the analysis was limited to measuring 5-HT levels without evaluating its metabolites (e.g., 5-hydroxyindoleacetic acid) or receptors, which restricts the ability to draw mechanistic conclusions. Second, the absence of systematic health assessments (e.g., infections or medication use), reliance on caregiver-reported diagnostic histories for TD children, and lack of standardized psychiatric screening tools may have introduced potential confounding factors. Third, although this was a national multicenter study, its cross-sectional design precludes establishing causal relationships between 5-HT and ASD, underscoring the need for future prospective studies. Finally, standardized dietary assessments were not feasible due to the large, multi-city sample size, limiting the ability to account for dietary influences on 5-HT levels. These limitations highlight the importance of incorporating more comprehensive biomarker analyses and longitudinal designs in future research to advance understanding in this field.

Altogether, the results of this study reveal that serum 5-HT levels are significantly higher in children with ASD children compared with TD children, and this increase is related to the severity and neuropsychological development associated with ASD, especially in boys. Additionally, research on the mechanisms of 5-HT in ASD and its influence on sex bias is necessary to elucidate the gaps in the research regarding the high prevalence of ASD and gender-specific phenotypic expression.

## Electronic supplementary material

Supplementary Material 1

## Data Availability

No datasets were generated or analysed during the current study.
