# Supplementary material for: Gender specific influence of serotonin on core symptoms and neurodevelopment of autism spectrum disorders: A multicenter study in China
Source: Child Adolesc Psychiatry Ment Health. 2025 Mar 29;19:35. doi: 10.1186/s13034-025-00892-7 (PMC11954278; doi:10.1186/s13034-025-00892-7)
Supplement: Supplementary file 1 — Supplementary Material 1 [file 13034_2025_892_MOESM1_ESM.docx]

| **Supplementary Table 1. The scores of SRS, CARS, and CNBS-R2016 scores among ASD boys and girls** | | | | |
| --- | --- | --- | --- | --- |
| **Item** | **Total (Mean±SD)** | **Boys (Mean±SD)** | **Girls (Mean±SD)** | ***P*** |
| **SRS scale scores** | n = 1177 | n = 966 | n = 211 |  |
| **Social awareness** | 11.44±3.29 | 11.38±3.31 | 11.70±3.18 | 0.201 |
| **Social cognition** | 18.03±4.56 | 17.99±4.62 | 18.17±4.25 | 0.612 |
| **Social communication** | 32.38±8.88 | 32.23±8.92 | 33.08±8.76 | 0.206 |
| **Social motivation** | 15.10±5.00 | 15.01±5.02 | 15.50±4.89 | 0.195 |
| **Autistic mannerisms** | 13.30±6.96 | 13.35±6.01 | 13.03±5.69 | 0.477 |
| **Total score** | 90.27±22.76 | 89.99±22.94 | 91.49±21.94 | 0.386 |
| **CARS scale scores** | n = 1178 | n = 975 | n = 203 |  |
| **Total score** | 33.23±6.97 | 33.29±6.83 | 32.95±7.59 | 0.537 |
| **CNBS-R2016** | n = 992 | n =811 | n = 181 |  |
| **Genernal quotient** | 62.61±18.96 | 62.80±18.73 | 61.77±20.03 | 0.510 |
| **Gross motor** | 78.71±21.43 | 78.68±21.22 | 78.81±22.40 | 0.945 |
| **Fine motor** | 61.11±19.84 | 61.38±19.71 | 59.88±20.40 | 0.359 |
| **Adaptive behavior** | 63.39±21.94 | 63.71±21.47 | 61.92±23.95 | 0.321 |
| **Language** | 53.48±25.87 | 53.94±25.86 | 51.42±25.89 | 0.236 |
| **Personal-social** | 56.39±21.02 | 56.29±20.81 | 56.83±21.94 | 0.757 |
| **Communication Warning Behavior** | 42.93±21.60 | 42.72±21.68 | 43.84±21.29 | 0.529 |
| **Abbreviations: SRS, Social Responsiveness Scale; CARS, Childhood Autism Rating Scale; CNBS-R2016, Children Neuropsychological and Behavior Scale-Revision 2016; ASD, autism spectrum disorder; SD, standard deviation.**  **Unpaired t-test was employed to compare the difference of SRS, CARS and CNBS-R2016 scores between ASD boys and ASD girls.** | | | | |


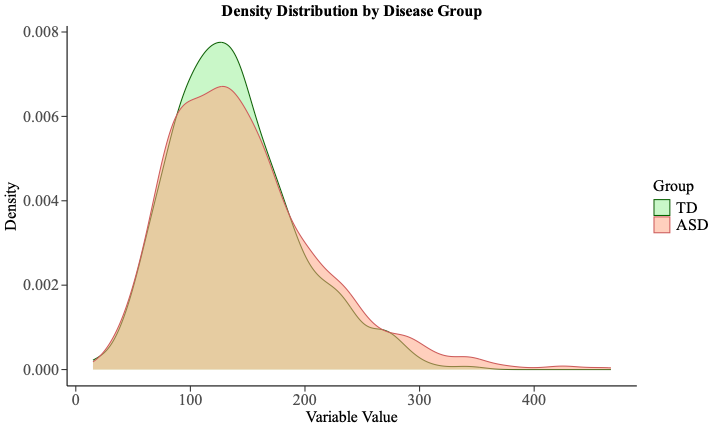


**Supplementary Figure 1. Density Distribution of 5-HT level in ASD group and TD group. ASD, autism spectrum disorder; TD, typically developing.**


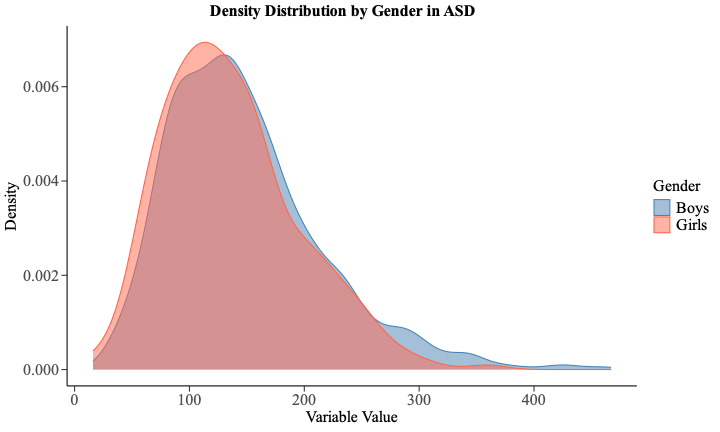


**Supplementary Figure 2. Density Distribution of 5-HT level in ASD boys and ASD girls. ASD, autism spectrum disorder.**
